# Differentiating Persistent Postural–Perceptual Dizziness from Anxiety Disorder Using Behavioral Responses to Complex Visual Environments

**DOI:** 10.3390/jcm15187082

**Published:** 2026-09-12

**Authors:** Kübra Orman, Hanifi Korkmaz, Sevilay Hançer Tecimer, Pınar Tekin, Sibel Çıplak, Hadeel Alsaleh, Monira I. Aldhahi

**Affiliations:** 1 Department of Psychiatry, Malatya Turgut Özal University, Malatya 44210, Türkiye; kubra.orman@ozal.edu.tr; 2 Battalgazi Vocational School, Malatya Turgut Özal University, Malatya 44210, Türkiye; hanifi.korkmaz@ozal.edu.tr; 3 Department of Otorhinolaryngology, Faculty of Medicine, Malatya Turgut Özal University, Malatya 44210, Türkiye; sevilay.tecimer@ozal.edu.tr; 4 Otorhinolaryngology, Malatya Training and Research Hospital, Malatya 44090, Türkiye; 5 Department of Neurology, Faculty of Medicine, Malatya Turgut Özal University, Malatya 44210, Türkiye; sibel.ciplak@ozal.edu.tr; 6 Department of Health Communication Sciences, College of Health and Rehabilitation Sciences, Princess Nourah Bint Abdulrahman University, P.O. Box 84428, Riyadh 11671, Saudi Arabia; hfalsaleh@pnu.edu.sa; 7 Department of Rehabilitation Sciences, College of Health and Rehabilitation Sciences, Princess Nourah Bint Abdulrahman University, P.O. Box 84428, Riyadh 11671, Saudi Arabia

**Keywords:** persistent postural–perceptual dizziness, anxiety disorder, virtual reality, vestibular assessment, visual dependence, differential diagnosis, sensory integration, avoidance behavior

## Abstract

**Background/Objectives:** Persistent postural–perceptual dizziness (PPPD) and anxiety disorder (AD) share overlapping clinical features that make differentiation with conventional assessments challenging. This study examined whether virtual reality (VR)-based vestibular assessment and immersive phobic scenarios can reveal distinct response profiles between these conditions. **Methods:** In this preliminary, single-center, cross-sectional study, 32 participants (PPPD: *n* = 15, AD: *n* = 17) underwent joint otorhinolaryngological/audiological and psychiatric evaluation, including the Clinical Test of Sensory Interaction and Balance-VR (CTSIB-VR), Limits of Stability (LOS), Dizziness Handicap Inventory (DHI), Beck Anxiety Inventory (BAI), and two immersive VR scenarios (CrowdVR and Supermarket Scrolling). Symptom onset latency, maximum tolerated intensity, avoidance behavior, and post-scenario Simulator Sickness Questionnaire (SSQ) scores were recorded after each scenario. **Results:** Groups did not differ on any LOS parameter. CTSIB-VR Visual Preference was significantly higher in the PPPD group (74.07 ± 7.44 vs. 60.82 ± 2.46, *p* < 0.001). SSQ Disorientation scores during the CrowdVR and Supermarket Scrolling scenarios were significantly elevated in PPPD (*p* = 0.004 and *p* = 0.002, respectively); at baseline VR exposure, SSQ Disorientation showed the same directional pattern but did not reach significance (*p* = 0.069), and the SSQ Total Severity score at baseline was numerically higher in PPPD (51.61 ± 17.62 vs. 41.13 ± 11.83) at a trend level (*p* = 0.055). BAI Total was markedly higher in the AD group (29.82 ± 4.86 vs. 19.27 ± 6.43, *p* < 0.001). Symptom onset latency during CrowdVR exposure was longer in PPPD (median 5.36 vs. 3.10 min, *p* = 0.007), whereas avoidance behavior was more pronounced in the AD group (*p* = 0.027). **Conclusions:** PPPD and AD showed divergent, exploratory VR-based response profiles: PPPD was marked by visual dependence, prolonged tolerance, and greater disorientation, while AD showed higher anxiety burden and earlier avoidance. These preliminary, hypothesis-generating findings suggest scenario-based VR assessment may merit further study as an adjunct in differentiating PPPD from anxiety disorder, pending diagnostic-accuracy validation in larger, multicenter cohorts.

## 1. Introduction

Persistent postural–perceptual dizziness (PPPD) is a chronic functional vestibular disorder, formally defined by the Bárány Society in 2017, that unifies the core features of several previously described clinical entities, including phobic postural vertigo, space–motion discomfort, visual vertigo, and chronic subjective dizziness [1,2,3]. With a prevalence of approximately 15–20% among patients presenting with vestibular complaints, PPPD is consistently identified as one of the most common diagnoses in both general neurology and dedicated vertigo outpatient clinics, and in some cohorts represents the leading diagnosis among working-age adults [3,4,5]. Clinically, the disorder is characterized by dizziness, unsteadiness, or non-spinning vertigo present on most days for three months or more, with symptoms typically exacerbated by upright posture, active or passive movement, and exposure to moving or visually complex environments [3].

Anxiety disorders represent the most frequent psychiatric comorbidity associated with PPPD and its predecessor syndromes, with reported co-occurrence rates of approximately 60% [6]. Conversely, dizziness and balance impairment are also commonly reported among individuals with primary anxiety disorders, particularly panic disorder and agoraphobia [7,8]. This substantial clinical overlap has led to ongoing diagnostic difficulty in differentiating PPPD from anxiety disorder, particularly given that physical examination and routine vestibular laboratory testing are typically unremarkable in both populations [2].

A central pathophysiological mechanism proposed to underlie PPPD is an alteration in multisensory integration, characterized by an increased reliance on visual input relative to vestibular and somatosensory cues for the maintenance of postural control, a phenomenon broadly termed visual dependence [9]. Neuroimaging investigations have provided convergent support for this model, demonstrating reduced functional connectivity between the precuneus and premotor cortical regions, alongside increased activity within visual cortical networks, in patients with PPPD relative to healthy controls [10,11]. Reduced precuneus-related connectivity has been associated with impaired regulation of posture and movement [12], while enhanced visual network activity is thought to underlie the excessive visual dependence observed in this population [10]. This shift toward visually weighted sensory processing is hypothesized to account for the characteristic exacerbation of dizziness and postural instability that patients with PPPD experience in visually dynamic, real-world environments such as crowded public spaces and supermarket aisles.

In parallel, functional neuroimaging studies comparing patients with PPPD and patients with primary anxiety disorders have identified both shared and distinct neural substrates. Both groups exhibit increased activation in threat-related regions, including the insula and inferior frontal gyrus, whereas patients with PPPD show greater activation in vestibulo-spatial regions such as the supramarginal and superior temporal gyri, suggesting a distinguishing role of vestibular-network dysfunction. Despite these neurobiological differences, psychometric questionnaires and routine vestibular testing have not consistently differentiated PPPD from primary anxiety disorder [13].

Virtual reality (VR) provides an opportunity to assess patients under visually dynamic conditions that better reflect symptom-provoking real-world environments. Previous work by Aharoni et al. demonstrated the feasibility of VR-based assessment in PPPD; however, comparisons were limited to healthy controls rather than patients with anxiety disorder. Consequently, it remains unknown whether VR-derived measures, including symptom onset latency, maximum tolerated stimulus intensity, and behavioral avoidance, can objectively distinguish these clinically overlapping populations [14].

Beyond its role in simulating ecologically relevant symptom-provoking environments, VR-based posturography offers specific methodological advantages over conventional static posturography: it enables controlled, reproducible manipulation of visual and somatosensory conditions and permits objective quantification of postural responses, allowing the relative contribution of visual, vestibular, and somatosensory systems to be estimated rather than directly isolated [15,16]. At the same time, VR-based balance assessment carries important limitations: substantial heterogeneity persists across studies in task conditions, sway parameters, and calculation methods, which constrains direct comparability of posturographic findings across platforms and populations, and standardized normative data for many VR-based paradigms, including scenario-based modules such as those used here, remain limited [17]. Continued technological refinement of headset resolution, motion-tracking accuracy, and standardized scoring algorithms is likely to further improve the reliability and cross-study comparability of VR-based vestibular and postural assessment [16,18].

The present study addressed this gap using VR-based vestibular assessment (CTSIB-VR) together with immersive real-world scenarios (CrowdVR and Supermarket Scrolling). We aimed to determine whether patients with PPPD and patients with anxiety disorder exhibit distinct response profiles beyond those detected by conventional psychometric and balance measures. We hypothesized that PPPD would be characterized predominantly by visual dependence and visually induced disorientation, whereas anxiety disorder would be characterized by greater anxiety burden and earlier behavioral avoidance. The primary outcomes were between-group differences in CTSIB-VR Visual Preference and SSQ-based clinical tolerance parameters, while DHI, BAI, and LOS were considered secondary/exploratory measures.

## 2. Methods

This study employed a cross-sectional, comparative observational design conducted at a single tertiary referral center. Two clinically defined diagnostic groups, namely patients with persistent postural–perceptual dizziness (PPPD) and patients with a primary anxiety disorder (AD), were compared on standardized audiovestibular, psychometric, and virtual reality (VR)-based vestibular and behavioral outcome measures, all administered within a single testing session per participant.

### 2.1. Ethical Approval

This study was approved by the Malatya Turgut Özal University Health Sciences Scientific Research Ethics Committee (Decision dated 25 July 2025, No. 2025/278). Data were collected between August 2025 and June 2026. All procedures were conducted in accordance with the ethical principles of the Declaration of Helsinki. Written informed consent was obtained from all participants prior to enrollment.

### 2.2. Participants and Setting

Participants were consecutively recruited from the Neurotology and Audiology Clinics of Malatya Training and Research Hospital (Malatya, Türkiye), a tertiary referral center for vestibular and balance disorders. Eligible individuals were allocated to one of two diagnostic groups on the basis of standardized clinical evaluation: patients meeting Bárány Society criteria for persistent postural–perceptual dizziness (PPPD), and patients meeting DSM-5 criteria for a primary anxiety disorder (AD), as detailed below.

### 2.3. Inclusion Criteria

Participants were eligible if they:

- Were between 18 and 60 years of age.
- Met the diagnostic criteria for Persistent Postural–Perceptual Dizziness (PPPD) established by the Bárány Society or fulfilled the diagnostic criteria for an AD determined by psychiatric evaluation.
- Reported persistent dizziness, non-spinning vertigo, and/or postural instability for at least 3 months, consistent with the minimum symptom-duration criterion specified in the Bárány Society consensus definition of PPPD [3].
- Were able to ambulate independently without assistive devices.
- Were able to understand and complete all study assessments and questionnaires.

### 2.4. Exclusion Criteria

Exclusion criteria included the following:

- Vestibular migraine according to the International Classification of Headache Disorders (ICHD-3).
- Acute vestibular disorders, including vestibular neuritis, labyrinthitis, active Ménière’s disease, or untreated benign paroxysmal positional vertigo.
- Patients with a confirmed psychiatric diagnosis of anxiety disorder in whom dizziness was reported solely as a somatic symptom without meeting the Bárány Society criteria for PPPD were allocated to the anxiety disorder group; individuals with anxiety disorder and concurrent dizziness who additionally fulfilled PPPD diagnostic criteria were excluded from both groups, as their inclusion would have precluded unambiguous group allocation.
- Neurological disorders affecting balance, such as Parkinson’s disease, multiple sclerosis, stroke, or cerebellar disorders.
- Significant musculoskeletal or orthopedic conditions impairing gait or postural control.
- Severe visual impairment not corrected by glasses or contact lenses.
- Severe psychiatric disorders (e.g., psychosis, bipolar disorder) or substance abuse.
- A history of epilepsy or other contraindications to virtual reality exposure.
- Inability to complete the assessment protocol.
- Current use of medications with known effects on vestibular or postural function (e.g., vestibular suppressants, benzodiazepines) or the presence of a comorbid psychiatric diagnosis beyond the primary study group classification.
- Panic disorder, with or without agoraphobia (excluded from the AD group a priori; see Section 2.8 below for rationale).

To minimize the potential effects of age-related vestibular decline, visual impairment, cognitive slowing, and adaptation difficulties associated with immersive virtual reality environments, individuals older than 60 years were excluded.

### 2.5. Recruitment and Screening Flow

Of 28 patients with PPPD assessed for eligibility, 15 were included in the final analysis; 13 were excluded (neurological disease, *n* = 4; active peripheral vestibular disorder, *n* = 5; unwillingness to participate in testing, *n* = 3; not suitable for testing due to a major psychiatric disorder, *n* = 1). Among the 15 included PPPD patients, the most common identified precipitant was an acute vestibular event (*n* = 7), followed by benign paroxysmal positional vertigo (*n* = 4) and vestibular neuritis (*n* = 2); no clear precipitant could be identified in 2 patients, one of whom had a history of migraine. Similarly, of 25 patients with a primary anxiety disorder assessed for eligibility from the psychiatry outpatient clinic, 17 were included in the final analysis; 8 were excluded (a coexisting history of a vestibular condition capable of confounding balance outcomes (benign paroxysmal positional vertigo, an episode of vertigo, or Ménière’s disease), *n* = 3; a neurological or orthopedic condition, comprising active migraine and a lower-back/leg orthopedic condition precluding stable standing on the force platform, *n* = 2; unwillingness to participate in VR testing or claustrophobia related to the headset, *n* = 2; and pregnancy, *n* = 1). Screening and exclusion details are summarized in Table 1.

### 2.6. Baseline Vestibular Screening

Prior to inclusion, participants underwent a brief vestibular screening consisting of a structured vestibular symptom history and bedside balance and vestibulo-ocular reflex assessments. The screening protocol included evaluation of spontaneous and gaze-evoked nystagmus, the Head Impulse Test, Romberg test, and tandem stance assessment. Individuals with clinical findings suggestive of an active or otherwise explanatory vestibular, neurological, or balance disorder were excluded; this exclusion targeted findings indicative of an active or explanatory vestibular pathology rather than the chronic dizziness that defines PPPD, since ongoing dizziness was itself an inclusion feature of the PPPD group. This procedure was performed to reduce the potential influence of subclinical vestibular confounders on postural outcomes [19]. Comprehensive instrumented vestibular testing (e.g., video head impulse testing, caloric testing) was not performed; these more provocative procedures were considered poorly tolerated in this population, as several participants expressed reluctance toward test conditions that could acutely provoke dizziness. Instead, all participants underwent a standardized bedside otoneurological examination that included positional testing for benign paroxysmal positional vertigo, head impulse testing, and assessment of nystagmus and skew deviation. Although these individual components correspond to the HINTS battery, HINTS has been validated specifically for patients presenting with acute vestibular syndrome and has not been established as a diagnostic or screening algorithm for chronic dizziness or PPPD [20]. Accordingly, this bedside examination cannot substitute for comprehensive instrumental vestibular assessment (e.g., video head-impulse testing, caloric testing), which was not performed in the present study.

All participants additionally underwent a standard otolaryngological examination. Formal audiometric testing was attempted for all participants; however, several participants declined pure-tone audiometry due to reluctance regarding the enclosed, quiet sound-booth testing environment. For these participants, tuning-fork tests (Rinne and Weber) were performed instead, together with a structured hearing-loss history; participants who declined both formal audiometry and tuning-fork testing, or who were found to have a hearing impairment on either assessment, were excluded from the study.

### 2.7. Diagnostic Criteria for PPPD

The diagnosis of PPPD was established according to the Bárány Society diagnostic criteria [3]. Participants were required to meet all of the following criteria:

1. Persistent non-spinning dizziness, unsteadiness, or vertigo lasting for at least 3 months.
2. Symptoms present on most days and exacerbated by: upright posture, active or passive motion, exposure to moving visual stimuli or complex visual environments.
3. Symptoms precipitated by a condition causing acute or episodic vestibular symptoms, neurological illness, psychological distress, or another balance-related event.
4. Symptoms causing significant distress or functional impairment.
5. Symptoms not better explained by another medical or psychiatric disorder.

### 2.8. Diagnostic Criteria for Anxiety Disorder (AD)

Participants in the AD group were evaluated by a psychiatrist through a structured clinical interview and met DSM-5 criteria for an anxiety disorder (e.g., generalized anxiety disorder or other non-panic DSM-5 anxiety disorder subtypes). Panic disorder, with or without agoraphobia, was excluded from the AD group a priori. Agoraphobia is specifically defined by DSM-5 criteria as marked fear or avoidance of two or more of the following situations, using public transportation, being in open spaces, being in enclosed spaces, standing in line or being in a crowd, or being outside the home alone [21], a symptom profile that maps closely onto the crowded and enclosed commercial environments simulated in the CrowdVR and Supermarket Scrolling modules (See Figures S1–S5). Moreover, panic disorder and agoraphobia have been specifically linked to vestibular dysfunction and movement-provoked symptomatology [7,8], raising the possibility of substantial diagnostic and symptomatic overlap with PPPD if included. Including participants with panic disorder/agoraphobia could therefore have confounded interpretation of avoidance behavior specifically within these two scenarios, reflecting either a general anxiety-related response or a pre-existing, diagnosis-specific avoidance phenotype already tied to similar situational triggers. This exclusion was intended to reduce that specific source of ambiguity; we acknowledge that panic disorder shows considerable clinical overlap with PPPD, and that its exclusion may limit the applicability of the present findings to real-world clinical populations in which panic disorder and PPPD frequently co-occur. The AD group was predominantly composed of participants with generalized anxiety disorder, with a smaller number meeting criteria for other non-panic DSM-5 anxiety disorder subtypes. They did not meet the Bárány Society criteria for PPPD, and no active vestibular pathology capable of explaining their symptoms was identified during neurotological assessment. To further characterize the severity of anxiety symptoms within this group, the Beck Anxiety Inventory (BAI) was administered to all participants; the mean BAI Total score in the anxiety disorder group was 29.82 ± 4.86, corresponding to the severe anxiety range according to established BAI scoring guidelines [22,23], indicating that participants in this group presented with clinically significant anxiety symptom burden at the time of assessment, consistent with the predominantly generalized anxiety disorder clinical profile described above.

### 2.9. Audiovestibular and Psychometric Assessment

All participants underwent a standardized assessment protocol comprising audiovestibular testing, psychometric questionnaires, and virtual reality (VR)-based vestibular and behavioral evaluation, as detailed below. The audiologist administering the CTSIB-VR, LOS, and VR scenario modules (including investigator-rated avoidance scoring) was blinded to participants’ diagnostic group assignment, which had been established independently by the otolaryngologist and psychiatrist prior to the audiological assessment. Emergency otorhinolaryngological, neurological, and psychiatric support was available throughout testing in the event of acute vestibular or psychiatric symptoms.

#### 2.9.1. Dizziness Handicap Inventory (DHI)

Perceived dizziness-related disability was assessed using the Dizziness Handicap Inventory (DHI), a 25-item self-report questionnaire comprising physical, functional, and emotional subscales, originally developed by Jacobson and Newman [24]. The Turkish version of the DHI, previously demonstrated to be valid and reliable, was used in the present study [25].

#### 2.9.2. Beck Anxiety Inventory (BAI)

Anxiety symptom severity was assessed using the Beck Anxiety Inventory (BAI), a 21-item self-report questionnaire developed by Beck et al. [22] to measure the severity of somatic and cognitive symptoms of anxiety. The Turkish adaptation, validity, and reliability study of the BAI was performed by Ulusoy et al., and the Turkish version was used in the present study [23].

#### 2.9.3. Simulator Sickness Questionnaire (SSQ)

Virtual reality-induced symptoms were assessed using the Simulator Sickness Questionnaire (SSQ), a 16-item instrument in which items contribute to three subscales (Nausea, Oculomotor, and Disorientation) together with a Total Severity score, originally developed by Kennedy et al. [1]. Subscale raw scores (the sum of the relevant item ratings) were converted using the standard Kennedy weighting coefficients (Nausea × 9.54, Oculomotor × 7.58, Disorientation × 13.92), and the Total Severity score was calculated as the sum of the three raw subscale scores multiplied by 3.74, following the original scoring algorithm. The Turkish version of the SSQ, previously validated for reliability and internal consistency (Cronbach’s α = 0.854) in a healthy Turkish sample, was used for questionnaire administration [26]. The Total Severity score have been recalculated from the raw subscale data using the standard Kennedy et al. [1].

#### 2.9.4. CTSIB-VR Assessment

Postural control and sensory organization were evaluated using the virtual reality-based Clinical Test of Sensory Interaction and Balance (CTSIB-VR; Virtualis, Montpellier, France), administered on the StaticVR posturography platform with a MotionVR headset. The CTSIB-VR protocol reproduces the six standard sensory conditions of the original CTSIB: (1) stable surface/eyes open, (2) stable surface/eyes closed, (3) stable surface/sway-referenced vision, (4) unstable surface (foam)/eyes open, (5) unstable surface (foam)/eyes closed, and (6) unstable surface (foam)/sway-referenced vision, the latter three conditions performed with an Airex-type foam block. For each condition, participants stood barefoot with the medial malleoli centered over the horizontal midline of the force platform. Three trials were performed for each sensory condition, and the trial yielding the highest performance score was retained for analysis. This approach was adopted to minimize the confounding influence of initial adaptation to the novel VR environment, which may artificially depress performance on the first trial; retaining the best-performing trial provides a more stable estimate of each participant’s actual sensory integration capacity under VR conditions [27]. We acknowledge that this best-of-three approach is non-standard relative to the mean-of-trials convention used in most posturography protocols and may introduce optimistic selection bias; per-trial raw data were not retained for the present dataset, precluding a post hoc mean-of-trials sensitivity analysis, and this is noted as a limitation. At the end of the assessment, individualized scores for the somatosensory, visual, and vestibular systems were calculated, together with a composite score and a visual preference (visual dependence) score, each compared against the system’s normative age-matched reference database. The CTSIB-VR Visual Preference score was calculated as the ratio of postural performance (equilibrium score) under sway-referenced visual conditions (conditions 3 and 6) relative to performance under eyes-closed conditions (conditions 2 and 5), Visual Preference = (Condition 3 + Condition 6)/(Condition 2 + Condition 5), consistent with the standard sensory-ratio framework derived from the Sensory Organization Test methodology underlying the CTSIB paradigm [28]. Following this convention, and consistent with its application on the same VR-based CTSIB platform in our concurrent work [29], a higher Visual Preference score is interpreted here as reflecting a greater relative reliance on visual input, including inaccurate visual input, for postural control (i.e., greater visual dependence) [30]. The manufacturer’s software (hCTSIB module; Virtualis, Montpellier, France; Software Version V2.0.8) computes an analogous ‘visual dependency score’ from head-oscillation-derived equilibrium data, compared against age-matched normative reference values [31].

#### 2.9.5. Limits of Stability (LOS) Assessment

Dynamic postural control was further assessed using the Limits of Stability (LOS) protocol on the StaticVR posturography platform with a MotionVR headset (Virtualis, Montpellier, France), which quantifies reaction time, movement velocity, endpoint excursion, maximum excursion, and directional control during voluntary weight-shifting tasks toward visually presented targets [32]. Participants were instructed to shift their center of pressure toward each of eight radially distributed targets, presented via the VR headset, as quickly and accurately as possible.

#### 2.9.6. Avoidance Score

Avoidance behavior during each VR scenario was rated by the examining audiologist on a 0–3 scale, defined a priori as follows: 0 = no hesitation or avoidance, scenario completed in full; 1 = verbal hesitation or discomfort expressed, but the scenario was completed; 2 = behavioral avoidance signs (e.g., attempting to remove the headset, requesting to sit down) that were overcome with encouragement, allowing completion; 3 = the scenario was abandoned or refused (e.g., headset removed, session terminated). Ratings incorporated both verbal report (patient-expressed reluctance) and observed behavior. Assessments were conducted by a team of four trained examiners (audiologists), following shared, standardized rating criteria; however, each session was rated by a single examiner, and independent dual-rating was not performed. Consequently, formal inter-rater reliability (e.g., Cohen’s kappa or intraclass correlation) could not be calculated, and this is acknowledged as a limitation.

#### 2.9.7. CrowdVR Module

Tolerance to immersive, crowd-based visual environments was assessed using the CrowdVR module (Virtualis, Montpellier, France; Q-REC-VI-147, Revision 01), a virtual reality application designed for the treatment and assessment of visual vertigo, scrolling syndrome, and agoraphobic/claustrophobic symptom provocation [33]. Participants stood upright wearing the MotionVR headset and were immersed in a crowd environment selected from among six available settings (Shop, Corridor, Shopping Center—Ground Floor, Shopping Center—1st Floor, Subway, and Pedestrian Crossing), with crowd density (1–100%) and virtual protection radius (0.1–0.8 m) adjustable in real time by the examiner. As the CrowdVR module does not provide a standardized end-of-session outcome score, clinical tolerance was operationally quantified using pre-defined parameters, comprising the maximum tolerated stimulus intensity (%), symptom onset latency (minutes), investigator-rated avoidance behavior (0–3 scale), and post-scenario SSQ Disorientation and Total scores. For all VR scenarios, symptom onset was operationally defined as the examiner-judged emergence of a new symptom (e.g., nausea, disorientation, imbalance) or a clinically noticeable worsening of the participant’s pre-existing chronic dizziness relative to their pre-scenario state, rather than the onset of symptoms in an otherwise asymptomatic individual; this determination relied on the examiner’s clinical judgment together with the participant’s verbal report, as no formal prespecified numeric worsening threshold (e.g., a fixed-point increase on a rating scale) was applied during data collection. Following the calibration procedure described below, the examiner initiated each CrowdVR session at the calibrated moderate starting intensity and subsequently titrated crowd density and/or virtual protection radius in real time during the session, using the software’s live session controls [33], progressively increasing provocative intensity until the participant reported symptom onset or the software’s maximum settings were reached (100% crowd density; 0.1 m protection radius, i.e., minimal dodge distance). The intensity value at the point of symptom onset or session termination was recorded as the maximum tolerated intensity. The CrowdVR scenario was delivered using the CrowdVR software module (version V1.2.8; Virtualis, Montpellier, France) does not generate an automated end-of-session outcome score [33]; consequently, symptom onset latency, maximum tolerated intensity, and avoidance behavior were recorded externally by the examiner rather than computed by the software.

#### 2.9.8. Supermarket Scrolling Module

Visually induced postural and symptomatic responses to a simulated dynamic visual environment were assessed using the Supermarket Scrolling module (Virtualis, Montpellier, France; Version V1.0, Class I Medical Device), administered on the StaticVR force platform with a MotionVR headset. Participants stood barefoot with the medial malleoli centered over the horizontal midline of the force platform and were immersed in a linearly scrolling supermarket aisle, and required to maintain postural control via right and left foot weight-shifting while avoiding visual obstacles. Session parameters, including scrolling speed (0–50 km/h), obstacle presence and spacing (5–50 m), and shelf filling density (0–100%), were configured at the Medium difficulty preset, corresponding to a moderate level of visual and postural challenge [34]. As with the CrowdVR module, the Supermarket Scrolling module does not provide a standardized clinical outcome score; clinical tolerance was therefore quantified using the same pre-defined parameters, namely maximum tolerated speed (km/h), symptom onset latency (minutes), investigator-rated avoidance behavior (0–3 scale), and post-scenario SSQ Disorientation and Total scores, independently of the distance, average speed, and obstacle-avoidance metrics natively reported by the device. Similarly, scrolling speed was initiated at the calibrated Medium-preset baseline and subsequently increased in real time during the session using the software’s live speed controls [34], until the participant reported symptom onset or the module’s maximum speed (50 km/h) was reached; the speed value at that point was recorded as the maximum tolerated speed. Consistent with the manufacturer’s own guidance to discontinue exposure at the first sign of symptoms (typically diaphoresis) [34], sessions were terminated at the earliest clinical indication of symptom onset or participant-reported discomfort.

#### 2.9.9. Calibration of VR Scenario Intensity

Standardized scenario-based virtual reality modules such as CrowdVR and Supermarket Scrolling have only recently become available for clinical use, and normative or consensus-based intensity parameters for these specific applications have not yet been established in the peer-reviewed literature. To address this gap and to ensure that the stimulus intensity employed in the present study was clinically appropriate and ecologically reproducible, preliminary trials were first conducted in a cohort of 15 healthy young volunteers (mean age approximately 20 years, university students without vestibular or psychiatric complaints). These preliminary trials were used to identify a moderate, well-tolerated stimulus intensity, corresponding to intermediate crowd density and virtual protection radius settings for CrowdVR and the Medium difficulty preset for Supermarket Scrolling, that avoided ceiling effects (excessive tolerance precluding group differentiation) and floor effects (premature symptom onset precluding adequate exposure duration) observed at the extreme ends of the available intensity range. The resulting moderate-intensity starting protocol was subsequently applied uniformly to all study participants, allowing between-group comparisons in maximum tolerated intensity to be attributed to differences in clinical response profile rather than to between-subject variability in initial stimulus exposure. This approach is consistent with methodological precedents in the PPPD literature that emphasize standardized, identical initial visual stimulation across participants to enable valid between-group comparison, such as the use of fixed-duration, standardized video scenarios in computerized visual vertigo assessment [35] and fixed-protocol optokinetic stimulation parameters in virtual reality-based vestibular intervention trials [36]. Representative screenshots of the CrowdVR and Supermarket Scrolling session-configuration interfaces, illustrating the full range of adjustable parameters described above, are provided in the Supplementary Materials for transparency [33,34]. The level of technical detail provided here for both protocols (software revision, exact adjustable parameter ranges, and session-level titration procedure) was intended to meet recently proposed reporting standards for extended reality-based clinical interventions, which have highlighted that the majority of published VR/XR intervention studies provide insufficient content and hardware description to permit replication [37]. The fixed, ascending-intensity sequence (Supermarket Scrolling followed by CrowdVR, described below) was adopted to standardize testing order across participants; we acknowledge that this design does not permit disentangling order/adaptation or carry-over effects from scenario-specific effects.

### 2.10. Procedure

**Figure 1:**
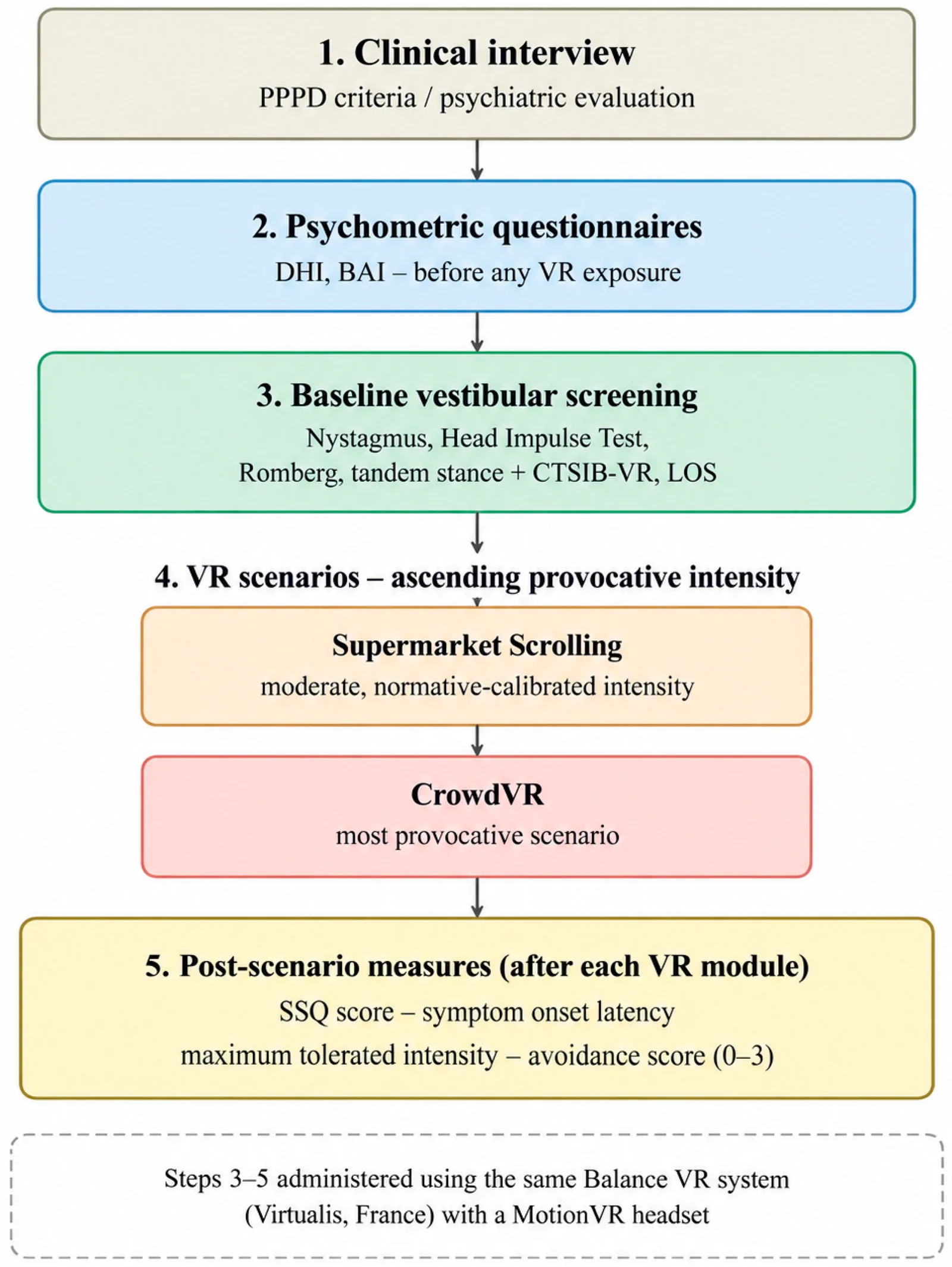
Schematic overview of the study procedure. Testing proceeded from clinical interview and diagnostic confirmation, through psychometric questionnaires administered prior to virtual reality exposure, baseline vestibular screening (bedside oculomotor and balance assessment together with CTSIB-VR and LOS), and virtual reality scenarios applied in ascending order of provocative intensity (Supermarket Scrolling followed by CrowdVR). Post-scenario clinical tolerance parameters (SSQ score, symptom onset latency, maximum tolerated intensity, and avoidance score) were recorded after each VR module. DHI: Dizziness Handicap Inventory; BAI: Beck Anxiety Inventory; CTSIB-VR: Clinical Test of Sensory Interaction and Balance-Virtual Reality; LOS: Limits of Stability; SSQ: Simulator Sickness Questionnaire.

All assessments were conducted at the Vestibular Assessment and Rehabilitation Clinic of Malatya Training and Research Hospital, in a single session for each participant, following a fixed sequence designed to minimize the influence of virtual reality-induced symptom provocation on subsequent measurements (Figure 1). First, participants underwent a preliminary otorhinolaryngological examination performed by an otolaryngologist, followed by a clinical interview and diagnostic confirmation, comprising application of the Bárány Society PPPD criteria and, for the anxiety disorder group, psychiatric evaluation performed by a psychiatrist according to DSM-5 criteria. Second, psychometric questionnaires (DHI and BAI) were administered by the psychiatrist prior to any virtual reality exposure, as post-exposure increases in anxiety or dizziness-related symptoms could otherwise confound questionnaire responses. Third, baseline vestibular and balance function were assessed by an audiologist, comprising the bedside vestibular screening protocol described above together with the VR-based CTSIB-VR and LOS protocols. Fourth, participants underwent the remaining VR scenarios, likewise administered by the (blinded) audiologist, in ascending order of provocative intensity, beginning with the Supermarket Scrolling module and concluding with the CrowdVR module, the latter considered the most visually and behaviorally provocative scenario. A rest period of approximately 5–10 min was provided between the Supermarket Scrolling and CrowdVR modules to allow symptoms provoked by the preceding scenario to subside before the subsequent exposure, consistent with general recommendations for minimizing carry-over effects between successive provocative sensory exposures in virtual reality-based assessment [34]. Following each VR scenario, the SSQ was administered together with the pre-defined clinical tolerance parameters (symptom onset latency, maximum tolerated stimulus intensity, and investigator-rated avoidance score). Steps 3–5 were performed using the same Balance VR system (Virtualis, Montpellier, France) with a MotionVR headset. No pre-exposure (baseline, before any VR scenario) SSQ measurement was obtained; consequently, post-scenario SSQ scores reflect cumulative exposure across the testing session rather than a change from a symptom-free baseline, and cannot be interpreted as purely scenario-specific symptom provocation. 

## 3. Statistical Analysis

Statistical analyses were performed using IBM SPSS Statistics (version 26.0; IBM Corp., Armonk, NY, USA). Normality of continuous variables was assessed using the Shapiro–Wilk test; normally distributed variables were compared using the independent-samples *t*-test and non-normally distributed variables using the Mann–Whitney U test, as reflected in the test labels provided in each table. Categorical variables were examined using the chi-square test. A two-tailed significance level of *p* < 0.05 was adopted for all analyses. Continuous variables are reported as mean ± standard deviation (Mean ± SD) or median (interquartile range; IQR) according to distribution.

As no standardized normative reference values or validated outcome measures have been established for the CrowdVR and Supermarket Scrolling modules, clinical tolerance was operationally quantified using pre-defined parameters comprising maximum tolerated stimulus intensity (%), symptom onset latency (minutes), investigator-rated avoidance behavior (0–3 scale), and post-scenario SSQ Disorientation and Total scores, each subjected to between-group statistical comparison. Symptom onset latency was recorded as a continuous value for all participants with no missing data; as no participant failed to reach a recorded onset value within the exposure window, time-to-event methods were not considered necessary for these comparisons, and the Mann–Whitney U test was retained for consistency with the other non-normally distributed outcomes.

No a priori power analysis was conducted, as the sample size was constrained by patient availability within a single clinical center during the recruitment period, a recognized challenge given the relatively low prevalence of PPPD. The present study is accordingly characterized as an exploratory group comparison rather than a diagnostic-accuracy study; given the number of simultaneous comparisons performed (approximately 30 univariate tests), a Benjamini–Hochberg false-discovery-rate (FDR) correction was applied across all comparisons [38]. Effect sizes are reported as Hedges’ g with 95% confidence intervals for independent-samples *t*-test comparisons (correcting for small-sample bias) and as rank-biserial correlation for Mann–Whitney U comparisons; both raw and FDR-adjusted *p*-values are reported. Despite the number of comparisons performed, the pattern of statistically significant between-group differences was largely preserved after FDR correction, which provides some reassurance against multiplicity-driven false positives; nonetheless, given the exploratory, single-center, pilot design of this study, all reported findings should be interpreted as hypothesis-generating rather than confirmatory.

## 4. Results

A total of 32 participants were enrolled in the study, comprising 15 individuals in the PPPD group (10 female, 5 male) and 17 in the AD group (11 female, 6 male). Age was not normally distributed in the PPPD group (Shapiro–Wilk *p* = 0.033) and was therefore compared using the Mann–Whitney U test; median age was 42.00 years (Q1–Q3: 31.00–44.50) in the PPPD group and 41.00 years (Q1–Q3: 38.00–42.00) in the AD group, with no statistically significant difference between groups (*p* = 1.000). Similarly, no significant difference was observed in sex distribution between groups (Fisher’s exact test, *p* = 1.000). Demographic characteristics of both groups are presented in Table 2.

The Simulator Sickness Questionnaire (SSQ) Nausea subscale did not differ significantly between groups (PPPD median 28.60, Q1–Q3 23.85–38.20; AD median 28.60, Q1–Q3 19.10–38.20; *p* = 0.266). The SSQ Disorientation subscale score was higher in the PPPD group (85.37 ± 30.62) than in the AD group (65.51 ± 29.01), but this difference did not reach statistical significance (*p* = 0.069). SSQ Oculomotor scores followed a similar pattern and likewise did not differ significantly between groups (*p* = 0.082). The SSQ Total Severity score, recalculated from the raw subscale data using the standard Kennedy et al. [1] scoring algorithm, was numerically higher in the PPPD group (51.61 ± 17.62) than in the AD group (41.13 ± 11.83), a difference that reached only a trend level of significance (*p* = 0.055, Hedges’ g = 0.69). In addition, DHI Total score was significantly higher in the PPPD group (40.53 ± 4.32) relative to the AD group (37.06 ± 3.60, *p* = 0.019). Significant differences were also observed for the DHI Functional (*p* < 0.001) and DHI Emotional (*p* = 0.004) subscales, both higher in the PPPD group, whereas the DHI Physical subscale did not differ significantly between groups (*p* = 0.615). As detailed in the Statistical Analysis, a Benjamini–Hochberg FDR correction was applied across all ~30 comparisons in this study; DHI Functional, DHI Emotional, and DHI Total remained significant after correction, whereas the SSQ Total Severity and Disorientation trend-level findings did not survive correction (Table 3). SSQ and DHI results are summarised in Table 3.

**Figure 2:**
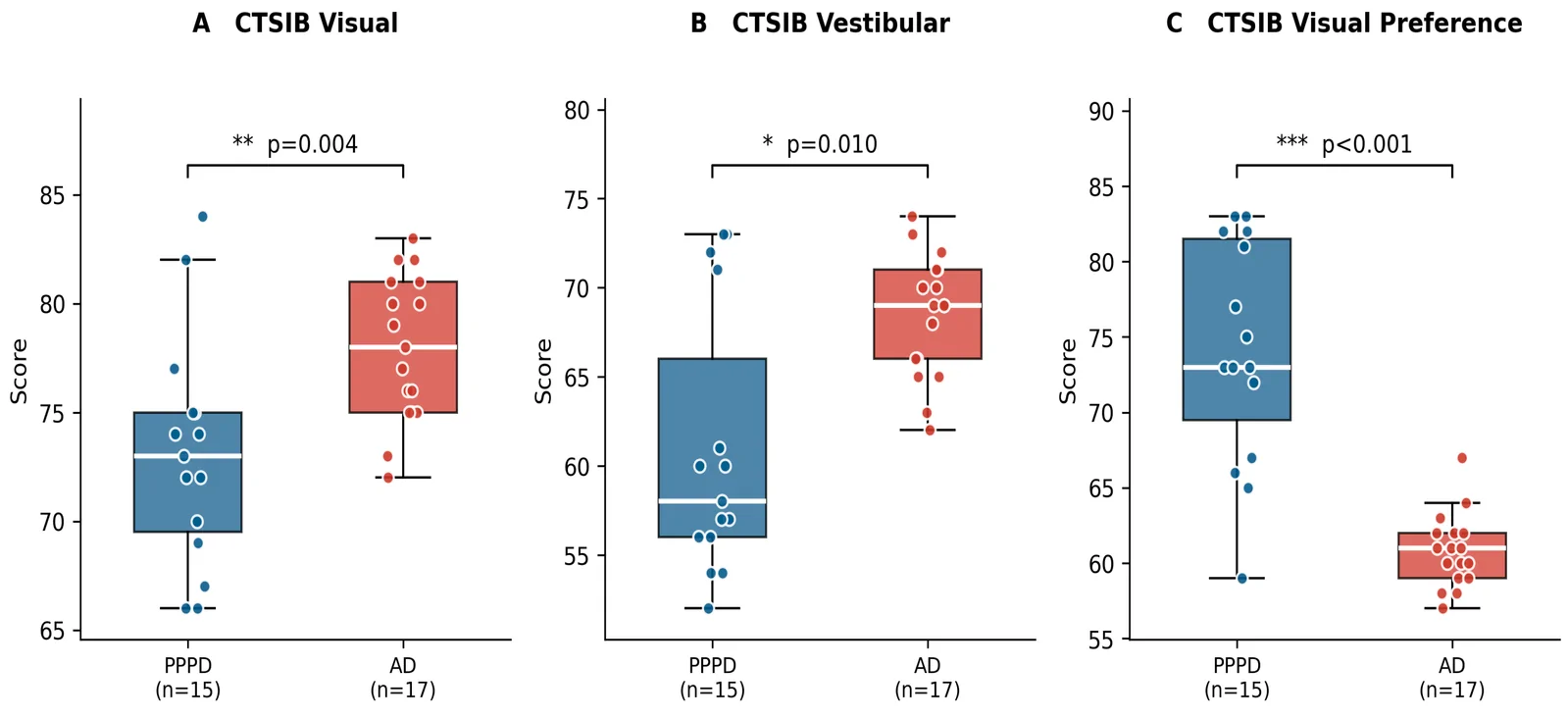
Between-group comparison of CTSIB-VR subscale scores. (**A**) CTSIB Visual scores were significantly higher in the anxiety disorder (AD) group compared to the PPPD group (*p* = 0.004). (**B**) CTSIB Vestibular scores were also significantly higher in the AD group (*p* = 0.010). (**C**) CTSIB Visual Preference scores were significantly elevated in the PPPD group relative to the AD group (*p* < 0.001), reflecting pronounced visual dependency. Box plots display median (white line), interquartile range (box), and 1.5 × IQR (whiskers); individual data points are overlaid. PPPD: Persistent Postural–Perceptual Dizziness (*n* = 15); AD: Anxiety Disorder (*n* = 17). * *p* < 0.05; ** *p* < 0.01; *** *p* < 0.001.

No statistically significant between-group differences were identified for any of the Limits of Stability (LOS) parameters, including Reaction Time (*p* = 0.925), Mean Velocity (*p* = 0.816), End Point Excursion (*p* = 0.325), Maximum Excursion (*p* = 0.970), and Directional Control (*p* = 0.308). CTSIB-VR assessment revealed significant group differences in several, though not all, sensory integration parameters. The CTSIB Visual score was significantly higher in the AD group (77.94 ± 3.36) compared to the PPPD group (73.07 ± 5.27, *p* = 0.004). Similarly, the CTSIB Vestibular score was significantly higher in the AD group (median 69.00) relative to the PPPD group (median 58.00, *p* = 0.010). Conversely, the CTSIB Visual Preference score was significantly higher in the PPPD group (74.07 ± 7.44) than in the AD group (60.82 ± 2.46, *p* < 0.001), indicating pronounced visual dependency in the PPPD group. The CTSIB Somatosensory subscale (*p* = 0.223) and CTSIB Composite score (*p* = 0.219) did not differ significantly between groups. All significant CTSIB-VR findings (Visual, Vestibular, and Visual Preference) remained significant after FDR correction. LOS and CTSIB-VR results are presented in Table 4. The between-group differences in CTSIB Visual, Vestibular, and Visual Preference scores are illustrated in Figure 2.

Beck Anxiety Inventory (BAI) scores were significantly elevated in the AD group across all subscales, all of which remained significant after FDR correction. The BAI Total score was 29.82 ± 4.86 in the AD group and 19.27 ± 6.43 in the PPPD group, with a highly significant between-group difference (*p* < 0.001). Significant differences were also identified for the BAI Somatic (*p* < 0.001) and BAI Cognitive (*p* < 0.001) subscales, with the AD group demonstrating markedly higher scores on both dimensions. BAI results are presented in Table 5.

**Figure 3:**
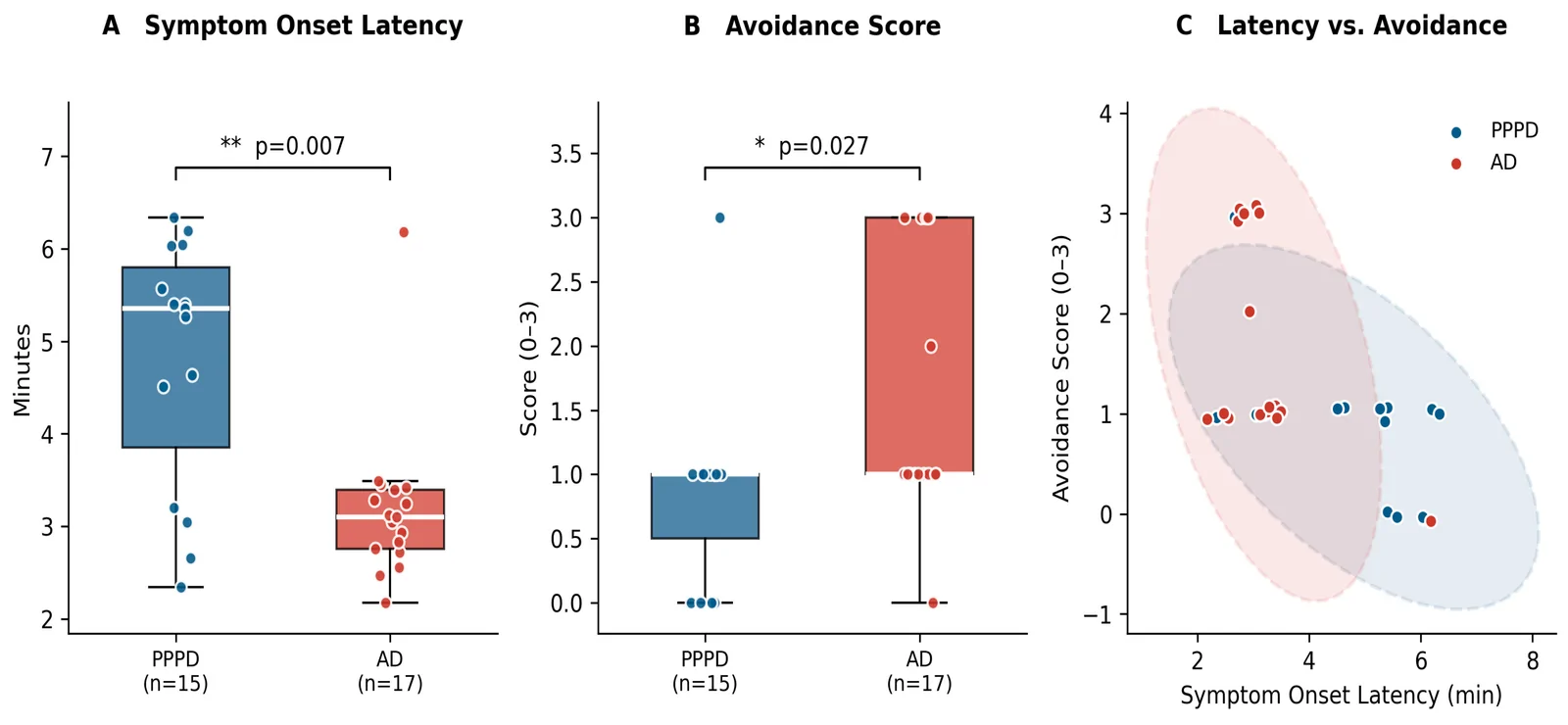
Between-group comparison of CrowdVR clinical tolerance parameters. (**A**) Symptom onset latency was significantly longer in the PPPD group (*p* = 0.007), indicating greater tolerance to immersive virtual crowd environments before symptom onset. (**B**) Avoidance behavior scores were significantly higher in the anxiety disorder (AD) group (*p* = 0.027), reflecting a tendency toward early behavioral avoidance. (**C**) Scatter plot of symptom onset latency versus avoidance behavior for individual participants, with dashed ellipses representing the approximate 95% data distribution for each group, illustrating the divergent, partially overlapping VR response profiles of the two clinical populations. PPPD: Persistent Postural–Perceptual Dizziness (*n* = 15); AD: Anxiety Disorder (*n* = 17). * *p* < 0.05; ** *p* < 0.01.

All CrowdVR clinical tolerance parameters demonstrated statistically significant between-group differences, all of which remained significant after FDR correction. The CrowdVR SSQ Disorientation score was significantly higher in the PPPD group (median 72.00) compared to the AD group (median 41.00, *p* = 0.004). Similarly, the CrowdVR SSQ Total score was significantly elevated in the PPPD group (85.53 ± 16.97) relative to the AD group (61.29 ± 9.99, *p* < 0.001). The maximum tolerated stimulus intensity was significantly higher in the PPPD group (median 71.00%) than in the AD group (median 42.00%, *p* = 0.003). Symptom onset latency was significantly longer in the PPPD group (median 5.36 min) compared to the AD group (median 3.10 min, *p* = 0.007), indicating that PPPD participants tolerated the immersive environment for a considerably longer duration before symptom onset. Avoidance behavior was significantly higher in the AD group (median 1.00, Q1–Q3: 1.00–3.00) relative to the PPPD group (median 1.00, Q1–Q3: 0.50–1.00, *p* = 0.027). These findings collectively suggest divergent VR response profiles between the two clinical groups. CrowdVR clinical tolerance parameters are detailed in Table 6, and the between-group differences in symptom onset latency and avoidance behavior are illustrated in Figure 3.

Supermarket Scrolling module clinical tolerance parameters demonstrated a comparable pattern of group differentiation; all parameters except Speed Tolerance remained significant after FDR correction. The Supermarket SSQ Disorientation score was significantly higher in the PPPD group (median 63.00) compared to the AD group (median 44.00, *p* = 0.002). The Supermarket SSQ Total score was also significantly elevated in the PPPD group (median 88.00) relative to the AD group (median 60.00, *p* = 0.019). Symptom onset latency was significantly longer in the PPPD group (median 4.73 min) than in the AD group (median 2.40 min, *p* = 0.002). Avoidance behavior was significantly higher in the AD group (median 2.00) compared to the PPPD group (median 1.00, *p* = 0.018). Speed tolerance did not differ significantly between groups (PPPD: 3.03 ± 0.53 km/h, AD: 2.85 ± 0.39 km/h, *p* = 0.299). Supermarket Scrolling clinical tolerance parameters are presented in Table 7.

## 5. Discussion

The present study aimed to determine whether patients with persistent postural–perceptual dizziness (PPPD) and patients with AD exhibit distinguishable, objectively quantifiable response profiles when assessed through virtual reality-based vestibular testing and immersive, ecologically relevant phobic scenarios. The principal findings indicate that the two groups did not differ on conventional dynamic balance parameters (LOS), but diverged on measures reflecting visual dependence (CTSIB-VR Visual Preference), VR-induced disorientation, which was significantly elevated in the PPPD group during the CrowdVR and Supermarket Scrolling scenarios but showed the same directional pattern only at a non-significant/trend level at baseline VR exposure (SSQ Disorientation, *p* = 0.069; SSQ Total Severity, *p* = 0.055), and behavioral tolerance parameters (symptom onset latency, maximum tolerated intensity, and avoidance behavior) derived from the CrowdVR and Supermarket Scrolling modules. Specifically, the PPPD group demonstrated greater visual dependence, longer tolerance to immersive stimulation, and higher disorientation scores, whereas the AD group demonstrated higher anxiety burden (BAI) and earlier behavioral avoidance. These findings collectively suggest that, despite substantial clinical overlap, PPPD and AD may be characterized by partially distinct response mechanisms, one weighted toward visuo-sensory dependence and the other toward anxiety-driven avoidance, that become more apparent under ecologically valid, visually dynamic conditions than under static laboratory testing alone.

### 5.1. Conventional Balance Measures and the Need for Ecologically Valid Assessment

Aharoni et al. investigated dynamic balance in individuals with PPPD using a virtual reality-based Four-Square Step Test under different visual loading conditions [39]. Although no significant group differences in overall task performance were observed, higher anxiety, perceived disability, and lower balance confidence were associated with altered movement strategies within the PPPD group. Similarly, the present study did not identify clear between-group differences in conventional dynamic balance parameters (LOS), whereas the PPPD group demonstrated greater visual dependence and higher VR-induced disorientation on the CTSIB-VR and scenario-based modules. Taken together, these findings may suggest that PPPD-related abnormalities are more evident in sensory integration processes and visually mediated symptom responses than in conventional balance outcome measures alone, lending support to the rationale for incorporating ecologically valid, visually complex assessment paradigms, such as the one employed here, when evaluating this population.

This interpretation is further reinforced by the work of De Vestel et al., who reported that patients with PPPD exhibited significantly higher visual dependence scores than patients with chronic dizziness without PPPD, whereas classical clinical balance tests showed limited discriminative value between the two groups [40]. The authors emphasized that visual dependence may represent one of the more distinguishing features of PPPD, a conclusion that appears consistent with the present finding of significantly elevated CTSIB-VR Visual Preference scores in the PPPD group relative to the AD group. Notably, De Vestel et al. assessed visual dependence using the Visual Vertigo Analogue Scale, whereas the present study employed an immersive, VR-based quantification (CTSIB-VR Visual Preference) together with behavioral responses to ecologically realistic scenarios (CrowdVR, Supermarket Scrolling); this methodological extension may offer a complementary, more dynamic perspective on the same underlying construct.

### 5.2. Visual Dependence as a Core Feature of PPPD

Increased visual dependence has long been considered a defining feature of PPPD, with earlier clinical and experimental work demonstrating that exposure to visually dynamic environments is associated with symptom exacerbation and an increased reliance of postural control on visual input [41,42]. The elevated CTSIB-VR Visual Preference and disorientation scores observed in the PPPD group of the present study appear broadly consistent with this body of evidence. Powell et al. further reported that patients with PPPD demonstrate heightened sensitivity across multiple sensory modalities, not limited to vision, and that this relationship, although partially mediated by anxiety, could not be fully accounted for by anxiety alone; visual discomfort and visual difficulty were found to explain a substantial proportion of symptom variance in PPPD. This observation may help contextualize the present finding that elevated visual dependence and VR-induced disorientation emerged in the PPPD group largely independently of the anxiety burden captured by the BAI, which was, expectedly, higher in the AD group. In this sense, the present results may be interpreted as consistent with a broader sensory hypersensitivity or overload model of PPPD, rather than a model in which visual symptoms are simply a secondary manifestation of anxiety.

The posturographic literature provides additional, more direct support for the CTSIB-VR findings. Söhsten et al. reported that patients with PPPD performed worse than both healthy individuals and patients who had recovered from acute vestibular syndromes across multiple Sensory Organization Test conditions, with the PPPD group showing the highest likelihood of abnormal sensory organization patterns [43]. In strong alignment with these findings, Morisod et al. [44] also demonstrated via computerized dynamic posturography that PPPD patients exhibit specific alterations in sensory weighting, characterized by a heightened susceptibility to visual–vestibular conflict conditions [44]. These findings appear compatible with the elevated Visual Preference scores observed in the PPPD group of the present study and may jointly suggest that sensory reweighting processes, specifically an overreliance on visual over vestibular and somatosensory inputs, could be disrupted in PPPD. It should be noted, however, that the present study did not identify a significant between-group difference in the CTSIB-VR Composite score (*p* = 0.219), which may indicate that this disruption is more clearly captured by domain-specific parameters, such as Visual Preference, than by global composite indices.

### 5.3. VR-Induced Disorientation and Vestibulo-Spatial Processing

The elevated SSQ Disorientation scores observed in the PPPD group during the CrowdVR and Supermarket Scrolling scenarios (though not reaching statistical significance at baseline VR exposure) may be considered in light of neuroimaging evidence suggesting altered vestibulo-spatial processing in this population. Storm et al. demonstrated that visual and vestibular motion perception is processed differently in patients with PPPD compared with healthy individuals, proposing that abnormal sensory-perceptual scaling mechanisms may be present in PPPD and that complex visual stimuli could heighten spatial uncertainty and anxiety in this population [45]. The elevated SSQ disorientation scores identified in the PPPD group of the present study during the two immersive scenario modules may be interpreted, with appropriate caution, as a behavioral correlate of these proposed perceptual processing differences. Storm et al. further noted that complex, dynamic visual environments, such as traffic or crowded settings, may be particularly likely to induce spatial uncertainty in PPPD; the CrowdVR and Supermarket Scrolling scenarios employed in the present study were similarly designed to simulate such real-world, visually complex environments, which may explain why between-group differences were most pronounced in these ecologically oriented modules rather than during the more static baseline VR exposure.

At the neurofunctional level, Maywald et al. compared patients with PPPD and patients with primary anxiety disorders using fMRI and reported that, although both groups exhibited broadly similar activation patterns within anxiety-related neural networks, the PPPD group showed additionally increased activation in the supramarginal gyrus and superior temporal gyrus, regions implicated in vestibulo-spatial processing [13]. In the present study, the PPPD group likewise demonstrated elevated visual dependence and VR-induced disorientation, while the AD group demonstrated a more prominent anxiety burden and earlier avoidance behavior. Considered together, these findings may suggest that PPPD is not merely a vestibular manifestation of anxiety, but could represent a distinct clinical profile additionally characterized by differences in vestibulo-spatial processing. This interpretation is broadly consistent with the conceptual framework proposed by Nigro et al. in their review of neuroimaging studies in PPPD, which describes the disorder as arising from a shift in the interaction among visuo-vestibular, sensorimotor, and emotional networks, favoring visual over vestibular input, rather than from anxiety-related mechanisms in isolation [46]. Within this framework, the comparatively preserved LOS performance alongside markedly elevated visual dependence and disorientation scores observed in the present PPPD group may reflect a relatively selective involvement of visuo-vestibular integration processes, rather than a generalized impairment of dynamic postural control.

### 5.4. Avoidance Behavior: A Potential Marker of Anxiety-Driven Response

In contrast to the visual dependence findings, behavioral avoidance during the CrowdVR and Supermarket Scrolling scenarios was more pronounced in the AD group, together with markedly shorter symptom onset latency and lower maximum tolerated stimulus intensity. Visual vertigo and phobic postural vertigo (both historical precursors of PPPD) have previously been associated with increased avoidance behavior and heightened perceived postural threat during exposure to moving visual stimuli [42]. In the present study, however, pronounced avoidance behavior and lower tolerance were observed predominantly in the AD group rather than the PPPD group. This pattern may suggest that, at least under the moderate-intensity VR exposure conditions employed here, behavioral avoidance could be more closely related to general emotional burden than to visual dependence per se, with the PPPD group instead demonstrating a tendency to tolerate prolonged visual exposure despite experiencing greater subjective disorientation. This distinction, between a tolerance-with-disorientation profile in PPPD and an avoidance-with-anxiety profile in AD, may itself represent a clinically meaningful point of differentiation, though it should be interpreted as hypothesis-generating given the exploratory nature of the present comparison.

### 5.5. VR Exposure as a Potential Confounding Factor

Because the VR environment used to assess participants was itself capable of provoking dizziness, discomfort, or anxiety, the extent to which the observed responses reflect the underlying clinical condition rather than a generic reaction to VR exposure warrants explicit consideration. Several observations partially mitigate this concern. First, the SSQ Nausea subscale, the subscale most closely associated with classic VR-induced motion sickness (vection-related sensory conflict), did not differ significantly between groups (*p* = 0.266), whereas between-group differentiation was concentrated in the Disorientation subscale and in behavioral/perceptual parameters (visual preference, latency, avoidance). This pattern suggests that the observed group differences are unlikely to be driven primarily by a generic VR-sickness effect common to all participants, and instead may relate more specifically to PPPD-associated perceptual and spatial processing [47]. Second, the CrowdVR module does not involve simulated self-locomotion (optic flow of forward movement); the participant remains stationary while other agents move around them, a configuration that may elicit less illusory self-motion than viewpoint-translating environments. By contrast, the Supermarket Scrolling module does involve forward-moving optic flow, a configuration more classically associated with vection-induced motion sickness; this asymmetry between modules should be considered when interpreting the corresponding results, and is noted as a limitation. Third, the exclusion criterion of ‘inability to complete the assessment protocol’ functioned as a safety stopping rule for participants with excessive VR intolerance, but did not eliminate the underlying confounding possibility at a methodological level.

### 5.6. Synthesis: PPPD as a Distinct Clinical Entity

Taken together, the present pattern of findings, elevated visual dependence and VR-induced disorientation in the PPPD group juxtaposed with elevated anxiety burden and avoidance behavior in the AD group, appears broadly aligned with the perspective articulated by Maywald et al. and Powell et al., who each, from different methodological vantage points, propose that PPPD cannot be fully explained by anxiety alone and instead reflects a more specific alteration in visual, vestibular, and emotional network interactions [13,42]. The relatively clear separation between the two groups on VR-derived clinical tolerance parameters, in the relative absence of differentiation on conventional balance measures (LOS) or on the DHI Physical subscale, may further support the proposition that ecologically valid, scenario-based VR assessment could offer complementary value alongside traditional psychometric and posturographic instruments in the exploratory characterization of chronic dizziness syndromes; establishing formal diagnostic or incremental diagnostic value would require dedicated diagnostic-accuracy analyses (e.g., ROC/AUC, sensitivity/specificity) that were outside the scope of this pilot comparison. We also note that the present comparison involved deliberately non-overlapping, ‘pure’ diagnostic groups (patients with comorbid PPPD and anxiety disorder were excluded from both groups), which may limit generalizability to the clinically common presentation in which the two conditions co-occur and differential diagnosis is most difficult; furthermore, exacerbation by complex visual environments is itself part of the Bárány Society diagnostic criteria for PPPD, so the use of CrowdVR/Supermarket-based responses as discriminating variables carries some inherent construct overlap with the criteria used to define the PPPD group, and this should be considered when interpreting the present between-group differences.

### 5.7. Clinical Implications: A Complementary Role in Differential Assessment

From a clinical standpoint, the present findings point toward a specific, complementary role for scenario-based VR assessment rather than a standalone diagnostic test. Patients presenting with chronic, non-specific dizziness are typically evaluated through standardized vestibular history-taking and psychiatric screening; however, a substantial proportion remain diagnostically ambiguous even after this stepwise workup, particularly when psychiatric comorbidity coexists with or mimics functional vestibular symptoms [48]. In such cases, the present data suggest that scenario-based VR assessment could be considered as an additional, targeted evaluation step when diagnostic uncertainty persists after conventional vestibular and psychometric assessment, rather than as a replacement for it. Practically, our findings suggest that clinicians could jointly examine the pattern across several concurrently observed parameters, namely visual preference, symptom onset latency, maximum tolerated intensity, avoidance behavior, and disorientation severity, rather than relying on any single measure in isolation: a profile combining prolonged tolerance, greater disorientation, and elevated visual dependence would be more consistent with PPPD, whereas a profile combining early avoidance, lower tolerated intensity, and predominant anxiety burden would be more consistent with a primary anxiety disorder. That is, the VR-based assessment appears to characterize not only the severity of symptoms but also the sensory and behavioral style of the response to a standardized visual challenge. We emphasize, however, that this proposed use is intended as a hypothesis-generating clinical heuristic rather than a validated diagnostic threshold: given the exploratory, single-center design and modest sample size of this pilot study, formal diagnostic-accuracy metrics (ROC/AUC, sensitivity, specificity, and cut-off values), together with test–retest reliability and multicenter replication, would be required before this approach could be recommended for individual-patient diagnostic use.

Beyond diagnostic classification, the divergent response profiles observed here may also be worth considering, purely as a hypothesis, in relation to treatment planning. A recent systematic review of randomized controlled trials in functional and anxiety-related dizziness reported that conventional vestibular rehabilitation therapy (VRT) is associated with small-to-moderate improvements in dizziness-related disability, that cognitive behavioral therapy (CBT) is associated more specifically with reductions in anxiety and dizziness-related catastrophic beliefs, and that combined VRT + CBT programs are associated with larger and more consistent improvements than either modality alone, particularly when anxiety appears prominent [49]. One tentative possibility, offered here as a hypothesis for future testing rather than a recommendation, is that a PPPD-like profile (pronounced visual dependence, prolonged tolerance, and disorientation with comparatively less avoidance) might align more closely with VRT-oriented strategies, whereas an anxiety-like profile (early avoidance, low tolerated intensity, and predominant anxiety burden) might align more closely with CBT-oriented, exposure-based intervention, with a combined VRT + CBT approach potentially relevant for patients showing a mixed profile. We want to be explicit that this is speculative, derived post hoc from a single exploratory comparison, and untested in the present sample; it would require dedicated, prospective intervention studies before it could have any bearing on treatment selection.

### 5.8. Methodological Rationale for VR-Based Tolerance Parameters

To our knowledge, no standardized outcome measures have yet been established for immersive VR crowd or supermarket paradigms in the assessment of PPPD or anxiety disorder. Rather than treating this as a methodological gap to be filled arbitrarily, the present study adopted a pragmatic clinical tolerance framework grounded in parameters that are well established in exposure-based virtual reality research more broadly. In the anxiety and phobia literature, the effectiveness of VR exposure is commonly indexed by subjective symptom burden alongside physiological or behavioral indicators of stress response; for instance, Renner et al. assessed both subjective symptom burden and heart rate variability during VR and in vivo exposure to fear-inducing narrow-room scenarios in anxious individuals, underscoring the broader methodological principle that effective evaluation of VR-based exposure requires the combined use of subjective and behaviorally anchored outcome parameters [50]. Within the PPPD and vestibular literature specifically, comparable scenario-based outcome metrics have not yet been reported, which limits the extent to which the present findings can be directly benchmarked against prior work using identical parameters. Nevertheless, the symptom onset latency, maximum tolerated stimulus intensity, avoidance behavior, and post-exposure SSQ scores employed here possess face validity and clear clinical relevance, and their application to a vestibular population represents a methodological extension of an approach already validated in the broader anxiety and exposure-based VR literature. Further validation studies will be required before these parameters can be considered standardized outcome metrics for VR-based PPPD assessment.

### 5.9. A Multidisciplinary Perspective on Chronic Dizziness

A distinguishing feature of the present work is its explicitly multidisciplinary design: diagnostic classification integrated joint otorhinolaryngological/audiological assessment (bedside vestibular screening, CTSIB-VR, LOS) with structured psychiatric evaluation (DSM-5-based diagnosis, BAI), conducted collaboratively by otolaryngologists, audiologists, and a psychiatrist within a single testing protocol. This integrated framework directly addresses a recurring difficulty in general clinical practice, namely that patients presenting with chronic dizziness are frequently triaged into either otoneurological or psychiatric care pathways without formal cross-disciplinary assessment, despite the well-documented overlap between PPPD and anxiety disorder. By quantifying both vestibular/visual (CTSIB-VR) and psychiatric (BAI) dimensions within the same VR-based paradigm, this study illustrates how objective, scenario-based markers could support exploratory differential characterization at the interface of neurotology and psychiatry, an approach with direct relevance for general and internal medicine practitioners who are often the first point of contact for patients with unexplained chronic dizziness.

### 5.10. Limitations

This study should be regarded as a preliminary, hypothesis-generating pilot investigation rather than a definitive comparative trial, and its findings should be interpreted accordingly. Several limitations of the present study should be acknowledged.

An error in the calculation of the SSQ Total Severity score was identified during revision and corrected using the standard Kennedy et al. [1] algorithm; the corrected comparison is a non-significant trend (*p* = 0.055) rather than the originally reported significant difference. This correction does not affect the CTSIB-VR, DHI, BAI, or clinical-tolerance findings, which were independently verified against the raw dataset.

The sample size (PPPD: *n* = 15; AD: *n* = 17) is modest and lacks a healthy control group, limiting statistical power, generalizability, and the precision of the reported effect sizes. Generalizability is further limited by our a priori exclusion of panic disorder from the AD group, undertaken to avoid confounding avoidance behavior in the CrowdVR and Supermarket Scrolling scenarios with a pre-existing avoidance phenotype (see Diagnostic Criteria for Anxiety Disorder).

Several aspects of the VR protocol constrain interpretation. No pre-exposure SSQ baseline was obtained, and latency, intensity, avoidance, and post-scenario SSQ scores share the same adaptive exposure procedure and are therefore not fully independent; symptom onset latency reflects worsening of pre-existing dizziness rather than onset in an asymptomatic individual. The fixed testing sequence (Supermarket Scrolling before CrowdVR) does not allow order or carry-over effects to be fully separated from genuine scenario differences, and test–retest reliability was not assessed, as repeating this demanding protocol was judged an excessive burden for this symptomatic population.

Several specific measures also have documented gaps: the bedside vestibular screening did not include video head impulse or caloric testing (poorly tolerated by this population), so residual vestibular dysfunction cannot be fully excluded; the CTSIB-VR best-of-three-trials convention may introduce optimistic bias, and per-trial data were not retained for a sensitivity analysis; the avoidance score was rated by a single examiner per session, precluding inter-rater reliability; and a comprehensive comorbidity and medication inventory was not systematically recorded for all participants. Finally, the cross-sectional design precludes causal inference, and normative data for the CrowdVR and Supermarket Scrolling modules do not yet exist; all findings should be regarded as hypothesis-generating pending replication in larger, multicenter studies.

### 5.11. Future Directions

Future research should aim to replicate the present findings in larger, multi-center samples that include a healthy control group, allowing the visual dependence and disorientation profile observed in PPPD, as well as the anxiety-driven avoidance profile observed in AD, to be benchmarked against normative responses. Longitudinal designs would further help clarify whether the VR-derived clinical tolerance parameters identified here are stable trait-like markers of PPPD or, alternatively, change over the course of vestibular rehabilitation or psychiatric treatment, which would have direct implications for their potential use as outcome measures. Given that standardized normative values for scenario-based VR modules such as CrowdVR and Supermarket Scrolling do not yet exist, dedicated validation studies, ideally incorporating dose–response calibration across a range of stimulus intensities, a pre-exposure SSQ baseline, and formal diagnostic-accuracy analysis (ROC/AUC, sensitivity, specificity), are needed before these parameters can be adopted as standardized clinical or research outcome metrics, or before any claim of diagnostic or incremental diagnostic value can be supported. Future studies should also formally evaluate test–retest reliability of the VR-based protocols, ideally using a reduced-burden design (e.g., a shorter reassessment limited to the CTSIB-VR module, or a longer inter-session interval) that minimizes repeated exposure to the most provocative scenarios in this clinically vulnerable population. Future studies may also benefit from incorporating physiological indices of autonomic arousal, such as heart rate variability, alongside subjective and behavioral measures, an approach that has shown utility in the broader VR exposure literature and may further enrich the characterization of divergent response profiles between PPPD and anxiety disorder. Finally, given the present findings of preserved LOS performance alongside altered visual dependence in PPPD, future work could examine whether VR-based interventions specifically targeting sensory reweighting and visual desensitization, rather than generalized dynamic balance training, are more effective in this population.

## 6. Conclusions

This pilot study suggests that patients with PPPD and patients with anxiety disorder, despite considerable clinical overlap and largely comparable performance on conventional dynamic balance testing, may show differing VR-based response profiles of visual dependence and behavioral tolerance to ecologically relevant, visually complex scenarios; whether such profiles carry diagnostic value remains to be formally established. Patients with PPPD demonstrated a response pattern characterized by elevated visual preference, prolonged tolerance, and pronounced disorientation, whereas patients with anxiety disorder demonstrated a pattern characterized by heightened anxiety burden and earlier behavioral avoidance. These findings appear consistent with the broader conceptualization of PPPD as a condition involving altered visuo-vestibular sensory integration rather than a phenomenon fully explained by anxiety alone, and may support further investigation of immersive, scenario-based virtual reality assessment as a potential adjunctive tool in the differential evaluation of chronic dizziness at the interface of neurotology and psychiatry. Given the exploratory nature and modest sample size of this preliminary study, these findings should be regarded as hypothesis-generating rather than confirmatory, warranting replication, including diagnostic-accuracy analyses, in larger, controlled, multicenter, and longitudinal investigations before any clinical or diagnostic recommendations can be made.

## Figures and Tables

**Table 1:** Screening, Exclusion, and Precipitant Characteristics for the PPPD and AD Groups.

Category	*n*
PPPD group	
Assessed for eligibility	28
Included in final analysis	15
Excluded (total)	13
Neurological disease	4
Active peripheral vestibular disorder	5
Unwilling to participate in testing	3
Not suitable due to major psychiatric disorder	1
PPPD precipitant (n = 15 included)	
Acute vestibular event	7
Benign paroxysmal positional vertigo	4
Vestibular neuritis	2
No clear precipitant identified	2
AD group	
Assessed for eligibility	25
Included in final analysis	17
Excluded (total)	8
Coexisting vestibular condition history (BPPV, vertigo episode, or Ménière’s disease)	3
Neurological or orthopedic condition	2
Unwillingness/claustrophobia regarding VR headset	2
Pregnancy	1

Note. AD = anxiety disorder; PPPD = persistent postural–perceptual dizziness; BPPV = benign paroxysmal positional vertigo. One of the two PPPD patients with no clear identifiable precipitant had a history of migraine. A structured, comprehensive comorbidity inventory beyond the above was not systematically recorded for all participants and is not reported.

**Table 2:** Demographic Characteristics of Study Participants.

Variable	PPPD (*n* = 15)	AD (*n* = 17)	*p*
Age, years	42.00 (31.00–44.50)	41.00 (38.00–42.00)	1.000 ^a^
Sex (Female/Male), *n*	10/5	11/6	1.000 ^b^

^a^ Mann–Whitney U test (non-normal distribution). ^b^ Fisher’s exact test. AD: Anxiety disorder.

**Table 3:** Between-Group Comparison of Simulator Sickness Questionnaire (SSQ) and Dizziness Handicap Inventory (DHI) Scores.

Variable	PPPD (*n* = 15)	AD (*n* = 17)	Test Statistic	*p*	Effect Size	*p* (FDR)
SSQ Nausea	28.60 (23.85–38.20)	28.60 (19.10–38.20)	U = 156.5	0.266	r_a_^b^ = −0.23	0.344
SSQ Oculomotor	30.30 (26.50–37.90)	22.70 (22.70–30.30)	U = 172.5	0.082	r_a_^b^ = −0.35	0.121
SSQ Disorientation	85.37 ± 30.62	65.51 ± 29.01	t(30) = 1.884	0.069	g = 0.65 [−0.04, 1.35]	0.107
SSQ Total Severity	51.61 ± 17.62	41.13 ± 11.83	t(30) = 1.995	0.055	g = 0.69 [−0.01, 1.39]	0.090
DHI Physical	12.00 (9.50–14.00)	10.00 (10.00–13.00)	U = 141.0	0.615	r_a_^b^ = −0.11	0.680
DHI Functional	16.00 (14.00–17.00)	12.00 (12.00–13.00)	U = 231.5	<0.001 ***	r_a_^b^ = −0.82	<0.001 ***
DHI Emotional	16.00 (14.50–16.50)	12.00 (11.00–14.00)	U = 204.0	0.004 **	r_a_^b^ = −0.60	0.010 **
DHI Total	40.53 ± 4.32	37.06 ± 3.60	t(30) = 2.481	0.019 *	g = 0.86 [0.15, 1.57]	0.035 *

Note. Values are Mean ± SD for normally distributed variables (independent-samples *t*-test), or Median (Q1–Q3) for non-normally distributed variables (Mann–Whitney U test). Effect sizes: Hedges’ g (95% CI) for *t*-test comparisons; rank-biserial correlation (r_a_^b^) for Mann–Whitney comparisons. *p* (FDR) = Benjamini–Hochberg-adjusted *p*-value across all ~30 comparisons in the study. SSQ = Simulator Sickness Questionnaire; DHI = Dizziness Handicap Inventory; PPPD = persistent postural–perceptual dizziness; AD = anxiety disorder. * *p* < 0.05; ** *p* < 0.01; *** *p* < 0.001. Highlighted rows: SSQ Nausea is newly reported.

**Table 4:** Between-Group Comparison of LOS and CTSIB-VR Parameters.

Variable	PPPD (*n* = 15)	AD (*n* = 17)	Test Statistic	*p*	Effect Size	*p* (FDR)
LOS Reaction Time (s)	0.41 (0.31–0.49)	0.41 (0.33–0.50)	U = 124.5	0.925	r_a_^b^ = 0.02	0.956
LOS Mean Velocity	1.44 ± 0.52	1.39 ± 0.65	t(30) = 0.234	0.816	g = 0.08 [−0.60, 0.76]	0.873
LOS End Point Excursion	26.13 ± 9.23	29.50 ± 9.75	t(30) = −1.001	0.325	g = −0.35 [−1.03, 0.34]	0.373
LOS Max Excursion	45.38 (36.56–46.75)	40.70 (36.38–48.89)	U = 126.0	0.970	r_a_^b^ = 0.01	0.970
LOS Directional Control	35.38 (29.38–41.06)	39.38 (37.62–41.12)	U = 100.0	0.308	r_a_^b^ = 0.22	0.367
CTSIB Composite	68.87 ± 4.31	70.53 ± 3.16	t(30) = −1.255	0.219	g = −0.43 [−1.12, 0.25]	0.300
CTSIB Somatosensory	82.27 ± 4.11	83.82 ± 2.92	t(30) = −1.246	0.223	g = −0.43 [−1.12, 0.25]	0.300
CTSIB Visual	73.07 ± 5.27	77.94 ± 3.36	t(30) = −3.157	0.004 **	g = −1.09 [−1.82, −0.36]	0.010 **
CTSIB Vestibular	58.00 (56.00–66.00)	69.00 (66.00–71.00)	U = 58.5	0.010 **	r_a_^b^ = 0.54	0.021 *
CTSIB Visual Preference	74.07 ± 7.44	60.82 ± 2.46	t(30) = 6.936	<0.001 ***	g = 2.40 [1.50, 3.29]	<0.001 ***

Effect sizes: Hedges’ g (95% CI) for *t*-test comparisons; rank-biserial correlation (r_a_^b^) for Mann–Whitney comparisons. *p* (FDR) = Benjamini–Hochberg-adjusted *p*-value across all ~30 comparisons. LOS = Limits of Stability; CTSIB-VR = Clinical Test of Sensory Interaction and Balance-Virtual Reality; AD = Anxiety Disorder. * *p* < 0.05; ** *p* < 0.01; *** *p* < 0.001.

**Table 5:** Between-Group Comparison of Beck Anxiety Inventory (BAI) Scores.

Variable	PPPD (*n* = 15)	AD (*n* = 17)	Test Statistic	*p*	Effect Size	*p* (FDR)
BAI Total	19.27 ± 6.43	29.82 ± 4.86	t(30) = −5.275	<0.001 ***	g = −1.82 [−2.63, −1.01]	<0.001 ***
BAI Somatic	12.00 (8.00–13.00)	15.00 (14.00–17.00)	U = 32.5	<0.001 ***	r_a_^b^ = 0.75	0.002 **
BAI Cognitive	8.07 ± 3.13	12.24 ± 3.23	t(30) = −3.697	<0.001 ***	g = −1.28 [−2.02, −0.53]	0.005 **

Effect sizes: Hedges’ g (95% CI) for *t*-test comparisons; rank-biserial correlation (r_a_^b^) for Mann–Whitney comparisons. *p* (FDR) = Benjamini–Hochberg-adjusted *p*-value across all ~30 comparisons. BAI = Beck Anxiety Inventory; AD = anxiety disorder. ** *p* < 0.01; *** *p* < 0.001.

**Table 6:** Between-Group Comparison of CrowdVR Clinical Tolerance Parameters.

Variable	PPPD (*n* = 15)	AD (*n* = 17)	Test Statistic	*p*	Effect Size	*p* (FDR)
SSQ Disorientation	72.00 (56.50–76.00)	41.00 (38.00–45.00)	U = 204.5	0.004 **	r_a_^b^ = −0.60	0.010 **
SSQ Total	85.53 ± 16.97	61.29 ± 9.99	t(30) = 4.994	<0.001 ***	g = 1.72 [0.93, 2.52]	<0.001 ***
Max Tolerated Intensity (%)	71.00 (55.50–76.50)	42.00 (34.00–45.00)	U = 206.0	0.003 **	r_a_^b^ = −0.62	0.010 **
Symptom Onset Latency (min)	5.36 (3.85–5.80)	3.10 (2.75–3.39)	U = 199.5	0.007 **	r_a_^b^ = −0.56	0.017 *
Avoidance Score (0–3)	1.00 (0.50–1.00)	1.00 (1.00–3.00)	U = 76.5	0.027 *	r_a_^b^ = 0.40	0.047 *

Effect sizes: Hedges’ g (95% CI) for *t*-test comparisons; rank-biserial correlation (r_a_^b^) for Mann–Whitney comparisons. *p* (FDR) = Benjamini–Hochberg-adjusted *p*-value across all ~30 comparisons. SSQ = Simulator Sickness Questionnaire; AD = anxiety disorder; CrowdVR = Virtualis, Clapiers, France (Q-REC-VI-147, Rev. 01). * *p* < 0.05; ** *p* < 0.01; *** *p* < 0.001.

**Table 7:** Between-Group Comparison of Supermarket Scrolling Clinical Tolerance Parameters.

Variable	PPPD (*n* = 15)	AD (*n* = 17)	Test Statistic	*p*	Effect Size	*p* (FDR)
SSQ Disorientation	63.00 (54.00–72.00)	44.00 (42.00–52.00)	U = 209.5	0.002 **	r_a_^b^ = −0.64	0.008 **
SSQ Total	88.00 (69.00–98.50)	60.00 (57.00–66.00)	U = 190.0	0.019 *	r_a_^b^ = −0.49	0.035 *
Speed Tolerance (km/h)	3.03 ± 0.53	2.85 ± 0.39	t(30) = 1.056	0.299	g = 0.36 [−0.32, 1.05]	0.367
Symptom Onset Latency (min)	4.73 (3.43–5.56)	2.40 (2.32–2.84)	U = 211.0	0.002 **	r_a_^b^ = −0.65	0.008 **
Avoidance Score (0–3)	1.00 (1.00–1.00)	2.00 (1.00–2.00)	U = 70.5	0.018 *	r_a_^b^ = 0.45	0.035 *

Effect sizes: Hedges’ g (95% CI) for *t*-test comparisons; rank-biserial correlation (r_a_^b^) for Mann–Whitney comparisons. *p* (FDR) = Benjamini–Hochberg-adjusted *p*-value across all ~30 comparisons. SSQ = Simulator Sickness Questionnaire; AD = anxiety disorder; Supermarket Scrolling = Virtualis, Montpellier, France (Version V1.0). * *p* < 0.05; ** *p* < 0.01

## Data Availability

The data presented in this study are available on request from the corresponding author due to privacy and ongoing-analysis restrictions.

## Supplementary Materials

The following supporting information can be downloaded at: https://www.mdpi.com/article/10.3390/jcm15187082/s1. Supplementary Figure S1. Representative CTSIB-VR results screen showing condition-specific balance scores, sensory analysis indices (Somatosensory, Visual, Vestibular, and Visual Preference), and composite performance metrics used to assess sensory organization and multisensory integration underlying postural stability. Supplementary Figure S2. CrowdVR environment and session settings. The figure illustrates the virtual crowd exposure interface, including crowd density and virtual protection radius parameters used to manipulate visual complexity and interpersonal distance during assessment. Supplementary Figure S3. CrowdVR subway environment. Additional environment-specific settings include station selection, exit-side manipulation, and adjustment of the number of virtual passengers to modify environmental complexity and crowd exposure intensity. Supplementary Figure S4. Supermarket Scrolling environment. Screenshot of the virtual supermarket aisle used in the Supermarket Scrolling module, illustrating the shelving, product displays, and promotional signage encountered during the low-to-moderate intensity VR exposure. Supplementary Figure S5. Supermarket Scrolling session settings. The figure illustrates the configurable session parameters (movement speed, obstacles and obstacle spacing, shelf filling, and tile scale) and preset difficulty levels (Very Easy to Extreme) used to calibrate module intensity.
