# Supplementary material for: Differentiating Persistent Postural–Perceptual Dizziness from Anxiety Disorder Using Behavioral Responses to Complex Visual Environments
Source: J Clin Med. 2026 Sep 12;15(18):7082. doi: 10.3390/jcm15187082 (PMC13607057; doi:10.3390/jcm15187082)
Supplement: Supplementary file 1 [file jcm-15-07082-s001.zip › jcm-4524497-supplemetary.pdf]

Supplementary Material

Differentiating Persistent Postural-Perceptual Dizziness from Anxiety Disorder Using Virtual Reality-Based Vestibular and Behavioral Assessment

2.1. Session settings

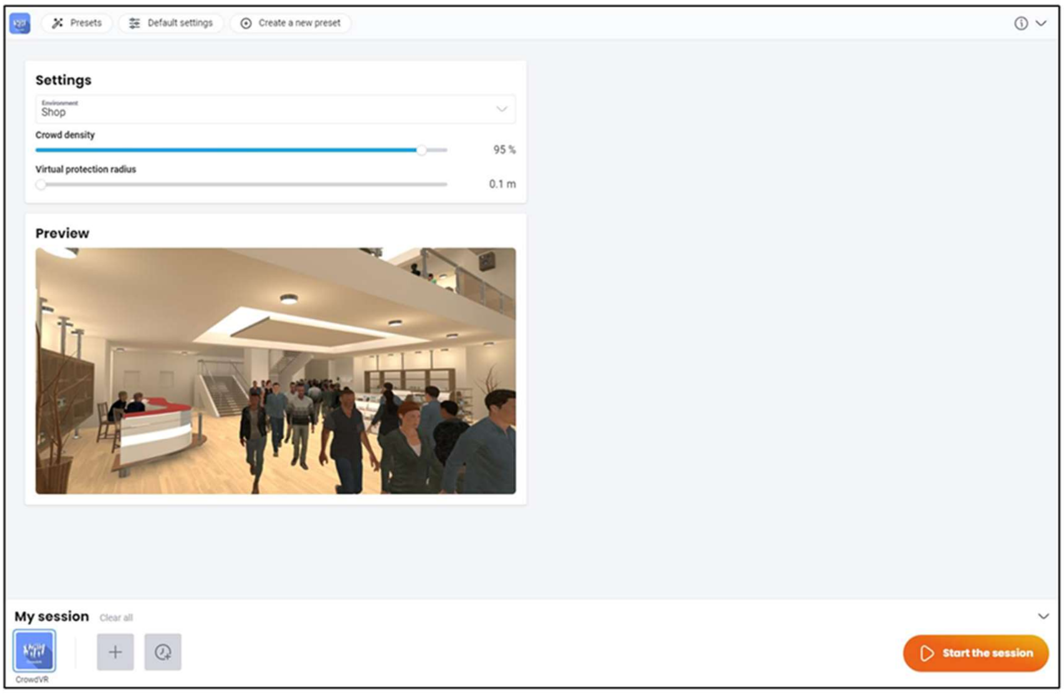

Supplementary Figure S1. Representative CTSIB-VR results screen showing condition-specific balance scores, sensory analysis indices (Somatosensory, Visual, Vestibular, and Visual Preference), and composite performance metrics used to assess sensory organization and multisensory integration underlying postural stability.

CrowdVR Module Tutorial

Revision 01
Date of issue: 18/06/2025

WARNING

The ability to move virtually without actually walking may cause sensory conflicts, leading to corresponding clinical signs (nausea). It is preferable to teleport.

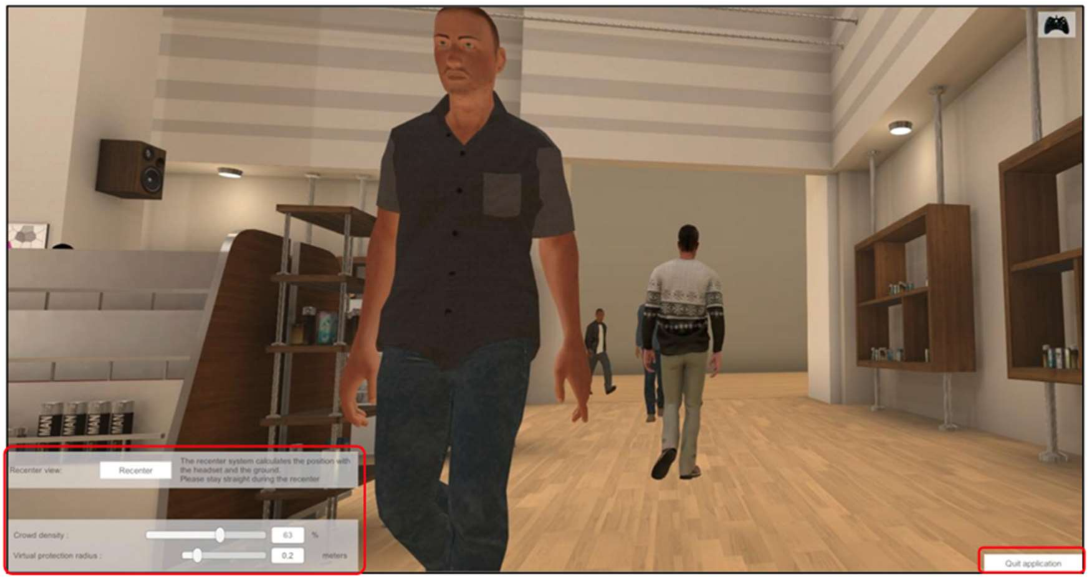

Supplementary Figure S2. CrowdVR environment and session settings. The figure illustrates the virtual crowd exposure interface, including crowd density and virtual protection radius parameters used to manipulate visual complexity and interpersonal distance during assessment.

3.1.1. Subway environment

The “Subway” environment includes additional settings:

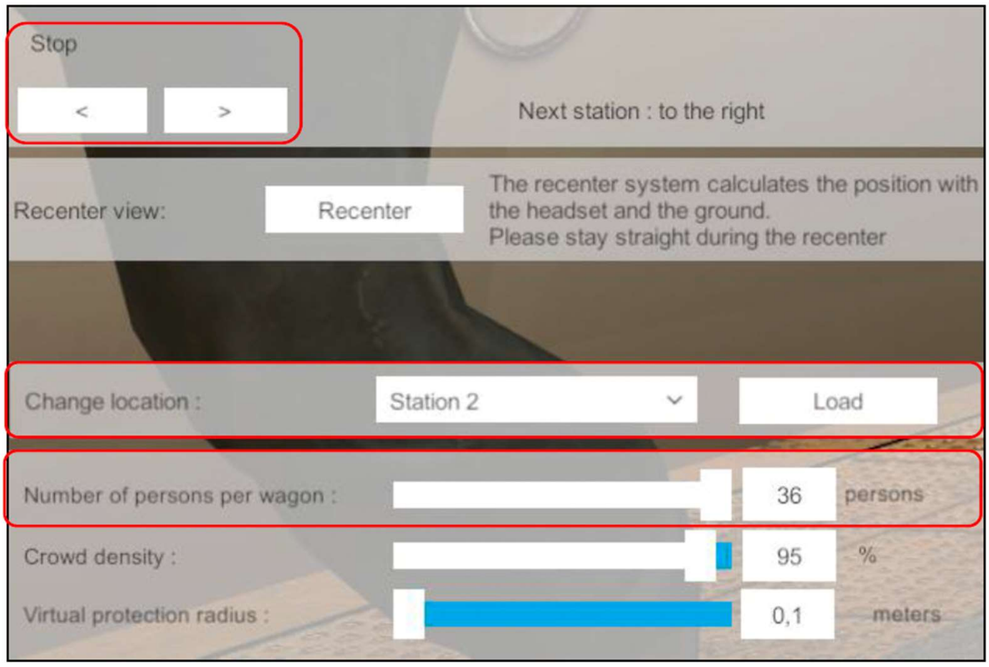

Supplementary Figure S3. CrowdVR subway environment. Additional environment-specific settings include station selection, exit-side manipulation, and adjustment of the number of virtual passengers to modify environmental complexity and crowd exposure intensity.

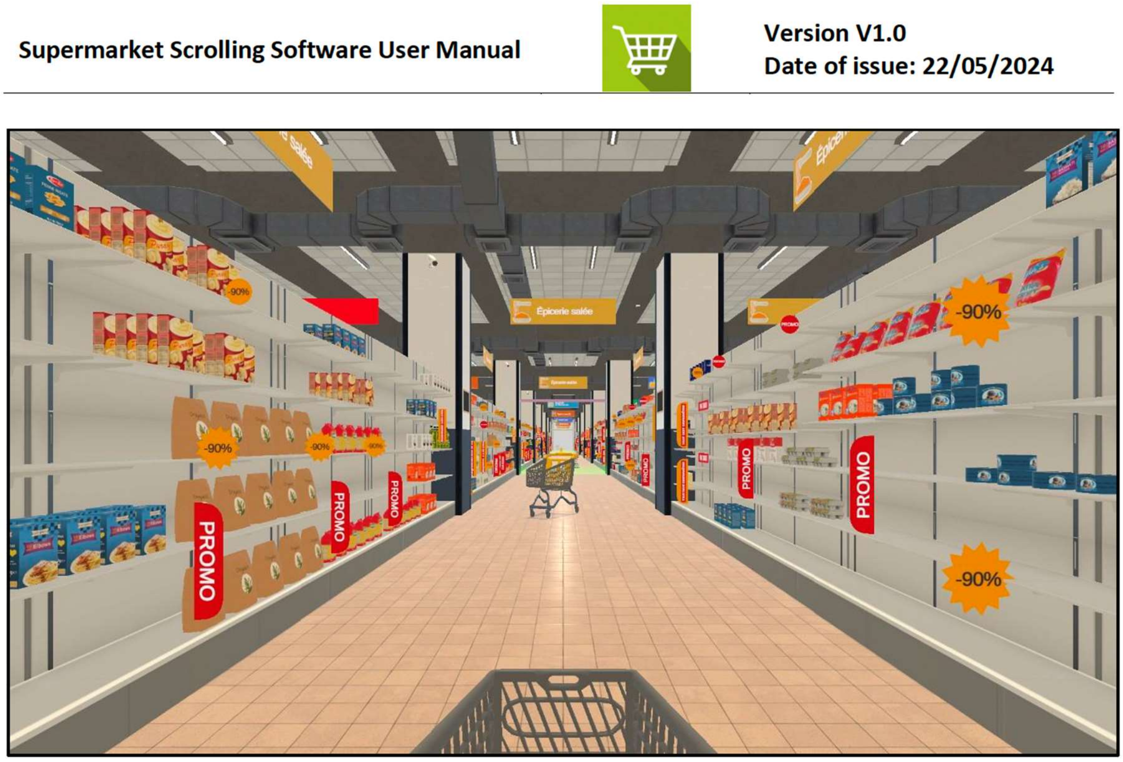

Supplementary Figure S4. Supermarket Scrolling environment. Screenshot of the virtual supermarket aisle used in the Supermarket Scrolling module, illustrating the shelving, product displays, and promotional signage encountered during the low-to-moderate intensity VR exposure.

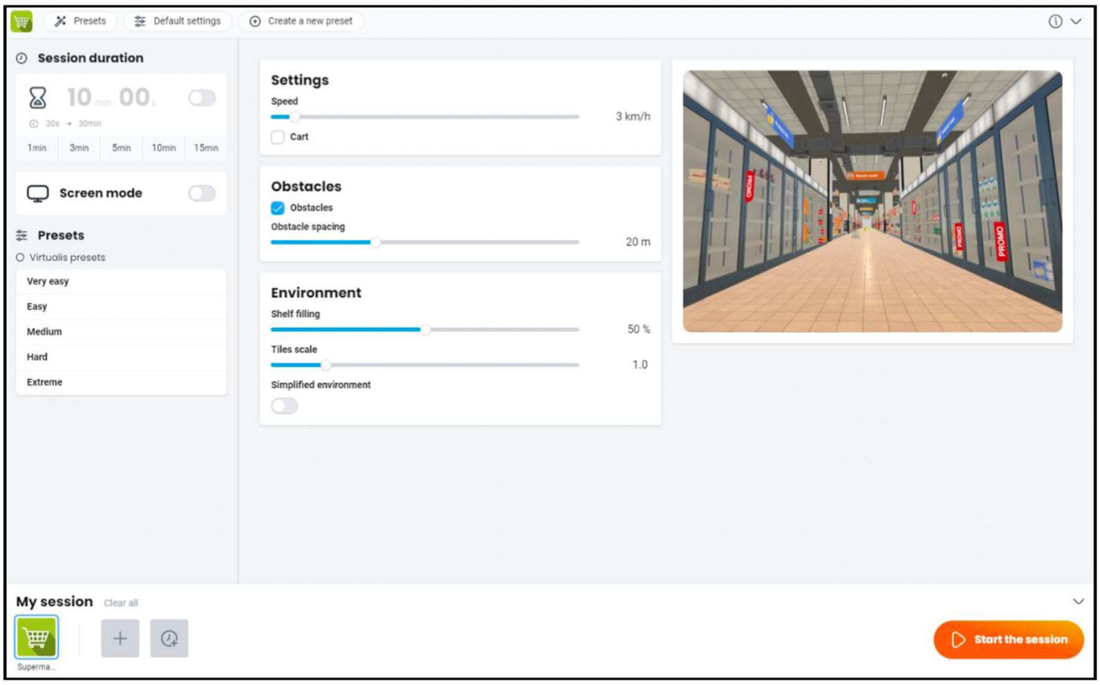

Supplementary Figure S5. Supermarket Scrolling session settings. The figure illustrates the configurable session parameters (movement speed, obstacles and obstacle spacing, shelf filling, and tile scale) and preset difficulty levels (Very Easy to Extreme) used to calibrate module intensity.
